# Investigation of Autostereoscopic Displays Based on Various Display Technologies

**DOI:** 10.3390/nano12030429

**Published:** 2022-01-27

**Authors:** Fuhao Chen, Chengfeng Qiu, Zhaojun Liu

**Affiliations:** 1 Department of Electrical and Electronic Engineering, Southern University of Science and Technology, Shenzhen 518055, China; chenfh@sustech.edu.cn (F.C.); qiucf@sitan.org (C.Q.); 2 Shenzhen Sitan Technology CO., LTD., 13F, Block A, Kaihaoda Plaza, Building 1, Industrial Park Road, Dalang Street, Longhua District, Shenzhen 518055, China

**Keywords:** autostereoscopic display, Micro-LED, light-emitting diode

## Abstract

The autostereoscopic display is a promising way towards three-dimensional-display technology since it allows humans to perceive stereoscopic images with naked eyes. However, it faces great challenges from low resolution, narrow viewing angle, ghost images, eye strain, and fatigue. Nowadays, the prevalent liquid crystal display (LCD), the organic light-emitting diode (OLED), and the emerging micro light-emitting diode (Micro-LED) offer more powerful tools to tackle these challenges. First, we comprehensively review various implementations of autostereoscopic displays. Second, based on LCD, OLED, and Micro-LED, their pros and cons for the implementation of autostereoscopic displays are compared. Lastly, several novel implementations of autostereoscopic displays with Micro-LED are proposed: a Micro-LED light-stripe backlight with an LCD, a high-resolution Micro-LED display with a micro-lens array or a high-speed scanning barrier/deflector, and a transparent floating display. This work could be a guidance for Micro-LED applications on autostereoscopic displays.

## 1. Introduction

Human eyes are capable of perceiving three-dimensional (3D) scenes and sensing the depth of objects, but the present two-dimensional (2D) displays are unable to show the depth perception, so people are pursuing more advanced 3D displays to make images closer to the reality. In physiology, depth cues include many agents; here, we focus on the physiological cues used in 3D displays: accommodation, convergence, binocular parallax, and motion parallax [1,2].

Accommodation refers to the adjustment of focal length of eyes on the watched object; convergence refers to the rotation of eyeballs to converge on the perceived point; binocular parallax, or binocular disparity, refers to the slightly different perceived images from left and right eyes, and the brain merges the two images into a stereoscopic image. It is the most important depth cue utilized in 3D displays. The last, motion parallax, refers to the relative location change of objects when moving our viewing position.

In these cues, binocular parallax gives rise to a strong depth sensation. Based on binocular parallax, many types of 3D displays were invented. In general, they can be classified into stereoscopic and autostereoscopic displays, respectively. Stereoscopic displays require audience wearing specialized glasses to perceive 3D images, but autostereoscopic displays permit watching 3D images with naked eyes. The very first autostereoscopic display was invented by Charles Wheatstone in 1830s using two tilted mirrors with 90 between them. Here, we focus on autostereoscopic displays because it is much closer to our natural visual experience. Moreover, in this article, the term “3D displays” is limited to “autostereoscopic displays”.

Autostereoscopic displays of interest to the market, and there are several commercial products which employ them, such as Nintendo 3DS, HTC EVO 3D, Sony Spatial Reality Display, and Google Starline. It shows that many corporations are striving to promote autostereoscopic displays to consumers. However, they still face challenges such as image blur, low resolution, narrow viewing angular range, limited viewing distance, eye strain, and fatigue [3,4].

## 2. Light-Field Displays

**Figure 1:**
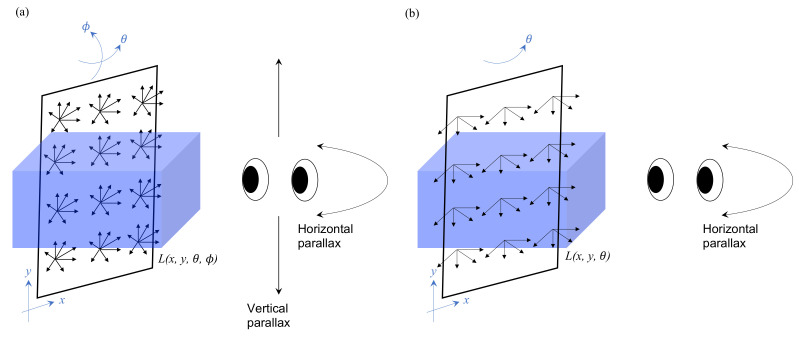
(**a**) A 4D light-field function L(x, y, θ, ϕ) fully presents the information of a 3D scene with a horizontal and vertical parallax. (**b**) To reduce the huge amount of information of a 4D light field, the ϕ-direction information is dropped and simplified into a 3D function L(x, y, θ), and only the binocular and horizontal parallax is preserved.

For autostereoscopic displays, the “light field” is a pivotal concept that must be mentioned. The term “light field” was coined by Andrey Gershun [5] in 1936. It illustrates the light intensity at a given position (x, y, z) and direction (θ, ϕ), and it is a 5D plenoptic function [6]. Furthermore, in 1996, Marc Levoy and Pat Hanrahan [7] proposed that the 5D function can be reduced to a 4D function as L(x, y, θ, ϕ) since the light ray remains unchanged along its propagation in free space. It implies that we can use a display that emits 2D spatial and 2D directional light rays to reproduce the light field including the depth information as shown in Figure 1a. Therefore, it fundamentally guarantees that 3D displays are feasible in principle, rather than a science fiction. However, the 4D function still carries too much information than the present technology can handle; to further reduce the information quantity, the vertical parallax depending on ϕ is dropped, since human perceives depth mainly based on horizontal binocular parallax of θ. Thus, the 4D function is reduced to a 3D function of (x, y, θ) as shown in Figure 1b. In general, 2D panels encode only the 2D spatial light intensity information but lack the light-ray directional information. However, it is possible to modify a 2D panel into a light-field display with light-directional-control elements, such as a parallax barrier [8,9], lenticular lens [10,11], and micro-lens array [12,13], etc.

## 3. Spatial Multiplex

Since the binocular parallax gives rise to a strong depth cue, people start to think how to make two eyes catch different perspective images. A parallax barrier or a lenticular sheet can achieve this. These methods usually redistribute the pixels evenly into two eyes, and this is called spatial multiplex. It is compatible with the modern liquid crystal display (LCD) or organic light emitting diode (OLED) display, but the drawback is that it reduces the resolution and luminance of the display. Commonly, spatial-multiplex displays are designed as two-view or multiple-view displays and multiple images share all the pixels evenly, so the spatial resolution of a single view is only 1/N, where N is the number of views. The problem can be resolved by the time-multiplex method, which is introduced in Section 4.

### 3.1. Parallax Barrier

**Figure 2:**
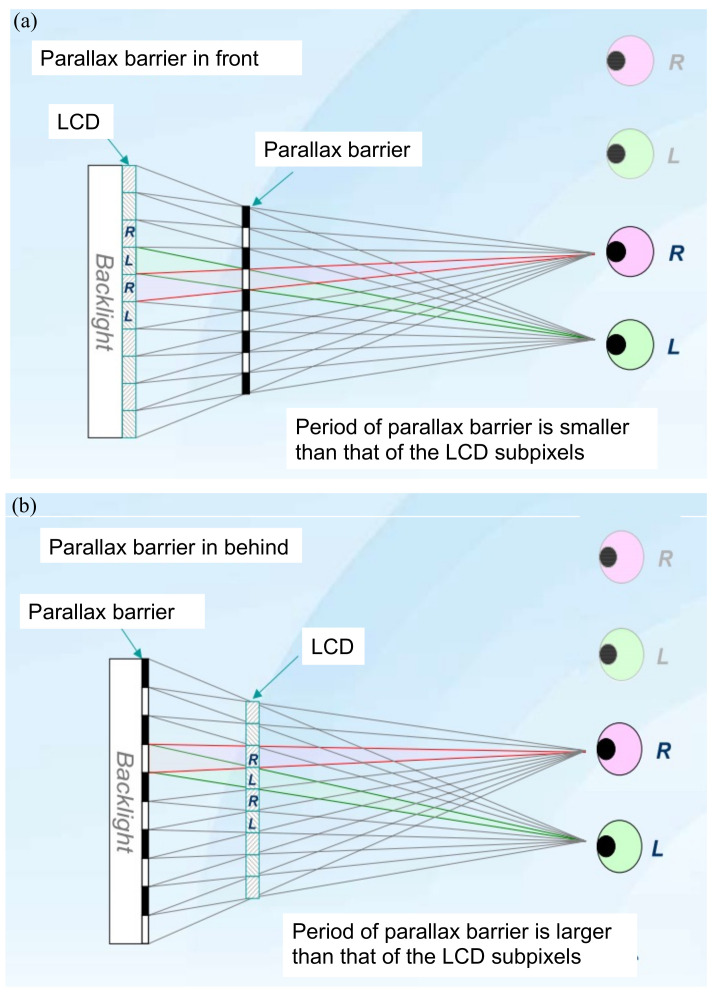
The parallax barrier is located (**a**) in front of the LCD panel or (**b**) behind the LCD panel. The parallax barrier generates the binocular parallax.

In 1896, Auguste Berthier [14] proposed a “parallax barrier” to create an auto-stereogram. Its mechanism is described in Figure 2, showing that the parallax barrier is (**a**) in front of the display panel or (**b**) behind the display panel. The parallax barrier is an interlace of transparent and opaque stripes. For the left eye, only the pixels labeled as L are perceived, and the pixels labeled as R are blocked. In the same way, only pixels labeled as R are perceived by the right eye. Thus, the left and right eyes watch different images, and it generates binocular parallax. In Figure 2b, the parallax barrier is inserted between the display panel and the backlight to make the barrier invisible, so people would not be aware of the existence of black strips. The details of the optical design can be found in Huang’s article [15].

**Figure 3:**
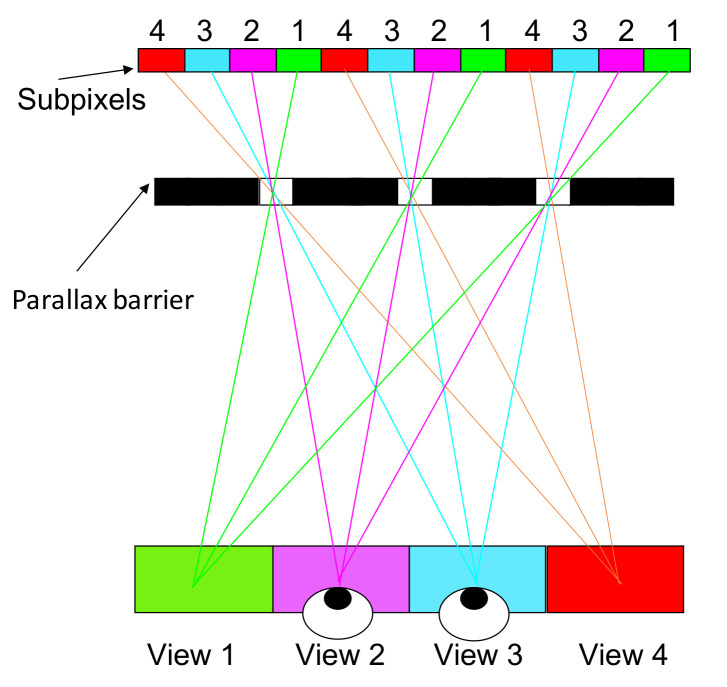
In the four-view design, the pitch of the parallax barrier is slightly smaller than four times the subpixel pitch.

The method can be modified into multiple views by changing the pitch of the barrier. Figure 3 demonstrates a four-view design, the pitch of the parallax barrier is slightly smaller than four times of the subpixel pitch to converge the light into a single view, and the width of the transparent region determines the brightness. Wider transparent region increases the brightness, but induces more crosstalk. A common width is one quarter of the pitch to avoid significant crosstalk. A multiview design offers a motion parallax on top of a binocular parallax, so it allows multiple people to move around and to watch 3D images. However, the brightness and horizontal resolution becomes only 1/N, where N is the view number. The low brightness remains a problem of the parallax barrier; in Section 3.2, a lenticular method is introduced to reconcile the brightness problem.

For practical use, this type of 3D display is usually designed as a 2D/3D switchable display. In real life, we have a bunch of 2D content such as texts, 2D images and videos, and watching 2D content with a parallax barrier makes the images fragmented, so switching back to the conventional 2D display is more suitable. The Nintendo 3DS [16] is a popular commercial product with a switchable parallax barrier. It used the configuration of Figure 2b and replaced the parallax barrier with a switchable liquid crystal (LC) shutter array, and it produced a two-view 3D image with 400 × 240 pixels for each eye. Sharp Inc. [17] also developed a 2D/3D switchable display with a switchable binary liquid crystal panel in front of a LCD; it is similar to the Nintendo 3DS but with a configuration shown in Figure 2a. Meanwhile, Samsung Inc. [18] and LG Inc. [19] have published a similar work with a switching LCD barrier inserted between the LCD and its backlight. Sanyo Inc. [20] used polymer-dispersed liquid crystal (PDLC) as a transparent/diffuse switchable film. With an applied electric field, the transparent mode makes the parallax barrier effective as a 3D mode; on the contrary, the diffuse mode disturbs the direction of light and makes the barrier ineffective as a 2D mode. It is worth mentioning that the Industrial Technology Research Institute (ITRI) [21] developed a localized 2D/3D switchable display and won the R&D100 award in 2010. It stacked two same-resolution LCDs and a micro-retarder film to form a localized parallax barrier, so the 2D content and 3D content were shown at the same time.

### 3.2. Lenticular Lenses

**Figure 4:**
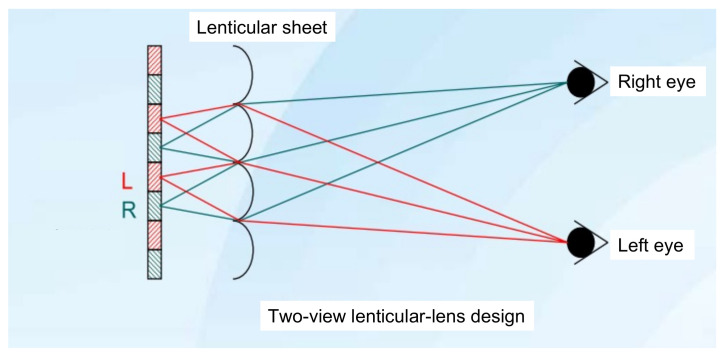
The lenticular-lens method used to produce a 3D image (top view) is plotted. It is similar to the parallax barrier; however, a lenticular sheet guides left-eye pixels to the left eye, and right-eye pixels to the right eye without blocking light.

The parallax-barrier method faces the degradation of brightness. To overcome the problem, Hess [22] invented a lenticular-lens method in 1915, which is described in Figure 4. The lenticular sheet is a one-dimensional cylindrical micro-lens array which replaces the role of the parallax barrier and collimates the diffused light from pixels into a specific direction, being received by a specific eye in the end. Hence, two eyes receive different images and merge them into an image with depth. Since the lenticular sheet is a transparent film, none of light is blocked; hence, it maintains the level of brightness. This is a remarkable advantage over a parallax barrier. On the other hand, a multiview setup can be implemented with the pitch of the lenticular array slightly less than N times of the width of a subpixel. However, it still suffers from the 1/N reduction in resolution.

Nowadays, many companies have developed lenticular 2D/3D switchable displays. Philips Inc. [23] developed LC switchable lenticular lenses that consist of a hollow lenticular shell filled LC in the hollow part. It is the 3D mode if the electric field is off, and 2D mode if the electric field is on. Given the mismatch of refractive index of LC and the material of lenticular shell, it behaves as a lens; on the contrary, with an electric field, the refractive index of LC would be the same as the lenticular shell, and the lens effect would vanish. Ocuity Inc. [24] developed another type of lenticular 2D/3D switchable display. The switchable lenticular lenses are comprised of a birefringent surface relief and a second layer made of isotropic material. It is polarization-sensitive, so a polarization rotator used to switch the polarization of light activates the function of lenticular lens in 3D mode. LG Inc. [25] adopted electric-field driven LC lenses as the switchable lenticular lenses. By controlling the distribution of the electric field, the LC has various rotation angles over the LC cell and forms a refractive index profile, equivalent to a convex lens. Without applying an electric field, it is nothing but a uniform-refractive-index plate, and behaves as a 2D mode. Chang et al. developed another configuration of rotatable 2D/3D display using an LC lens array with a gradient electric field to watch 3D content in landscape/portrait mode [26]. Lastly, ITRI demonstrated a lenticular sheet and a polymer dispersed liquid crystal (PDLC) inserted between an LCD panel and its collimated backlight. When PDLC is in clear mode, the lenticular sheet directs the light into viewing zones as the 3D mode; when the PDLC is in diffusing mode, the collimated backlight is diffused and behaves as the 2D mode [27].

Besides the multiview displays, a “super-multiview display” means a very high dense of view number that allows at least two views impinging into a single eye. Two or more views represent two or more rays entering the pupil of eyes such that eyes can focus on the intersection of two or more rays [28]. Researchers have demonstrated 64-, 72-, 128-, and 256-view systems with a micro-lens array [29,30,31,32,33,34]. The National Institute of Information and Communications Technology (NICT) [35] developed a super-multiview display with 57 units of projectors, and a viewing angle of 13. ITRI [36] also demonstrated a multiple projector systems involving 60 views and a 32-inch lenticular screen. Takaki et al. [37] proposed a tabletop 360 system with high-speed projectors and a rotating transmissive screen. However, super-multiview suffers a huge loss of resolution, so introducing a head-tracking system is an effective manner to keep the resolution from dropping too much. Sony Inc. [38] has developed a 15.6${}^{\prime \prime }$ 4 K light-field display with lenticules and a head-tracking system to avoid the pseudoscopic image and to reduce the resolution loss, but it is designed for a single user only.

## 4. Time Multiplex

The main shortcoming of the spatial multiplex is the severe drop in resolution, especially for multiview displays. To overcome the problem, “time multiplex” was proposed to solve it. The idea is multiplying the refresh rate by the number of views and reusing each pixel multiple times to compensate for the loss of resolution. This method projects each view time-sequentially. As an example of a two-view display, the refresh rate needs to be 60 Hz × 2 = 120 Hz to ensure that both eyes perceive images without flickers. Nonetheless, achieving an even higher refresh rate is not easy for common LCDs and the directional device, and it is also one of the major barriers to pursuing multiviews in time multiplex.

**Figure 5:**
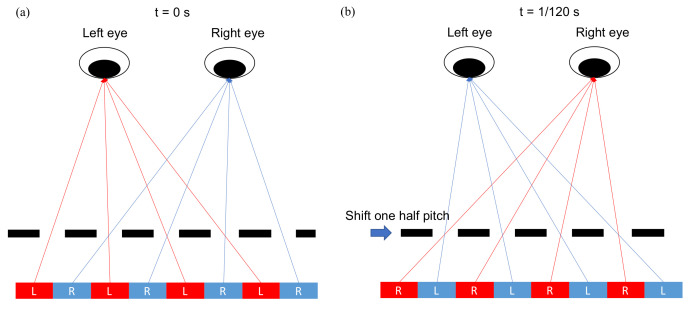
The parallax barrier slits are shifted by one half pitch within 1/120 s, so both eyes can perceive red and blue pixels, and then the resolution is retained. (**a**) As t = 0 s, the parallax barrier is at the original place. (**b**) As t = 1/120 s, the parallax barrier shifts one half pitch.

In general, the spatial multiplex can be generalized to time multiplex. For a parallax barrier, we can just shift the position of open slits by one half pitch, so all the pixels are perceived by two eyes, illustrated in Figure 5. In this case, the barrier and frame rate are scanning with doubled 120 Hz, so human eyes would not feel flickers.

This type of scanning parallax barrier can be realized with an electro-controllable LC shutter array. So far, most time-multiplex displays are two-view displays owing to the slow scanning rate of LC. Samsung Inc. [39,40] has developed a fast-scanning parallax barrier with an optical compensated bend (OCB) display mode to achieve a 5 ms response time, and an active-matrix OLED (AMOLED) display was adopted because of its high refresh rate. Overall, a 2.2-inch, full resolution 240 × 320, two-view time-multiplex display was demonstrated. PolarScreens Inc. [41] demonstrated a 120Hz 3D LCD panel, a vertical patterned active shutter panel and a head tracking system to achieve a full resolution autostereoscopic display.

**Figure 6:**
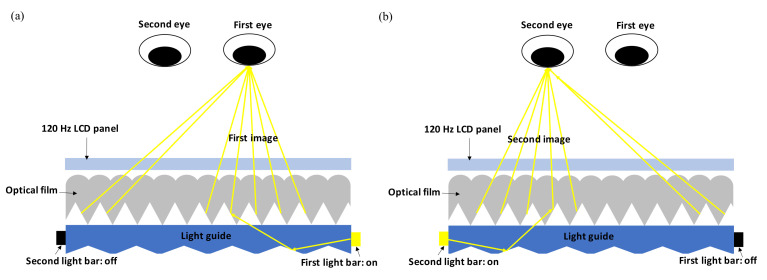
(**a**) The first light bar is turned on and projects the first image to the first eye via the guide of the optical film. (**b**) Similarly, the second light bar projects the second image to the second eye. The two light bars are turned on and off alternatively. (Reproduced with permission from ref. [42]).

Another configuration is using an edge-lit backlight with a special-designed optical film, described in Figure 6. The backlight has two light bars attached on each edge of the light guide; the optical film comprises of prism and lenticular structure which deflects rays to the specific direction. When the first light bar is turned on, the first image is projected to the first eye. In the same way, when the second light bar is turned on, the second image is projected to the second eye. The light bars are turned on and off alternatively and make two eyes perceive different images with full resolution. 3M Inc. [42] has developed an optical film, Vikuiti, that controls the directional function. On the other hand, AU Optronics Inc. [43] and Philips Inc. [44] have published patents with similar optical structures.

**Figure 7:**
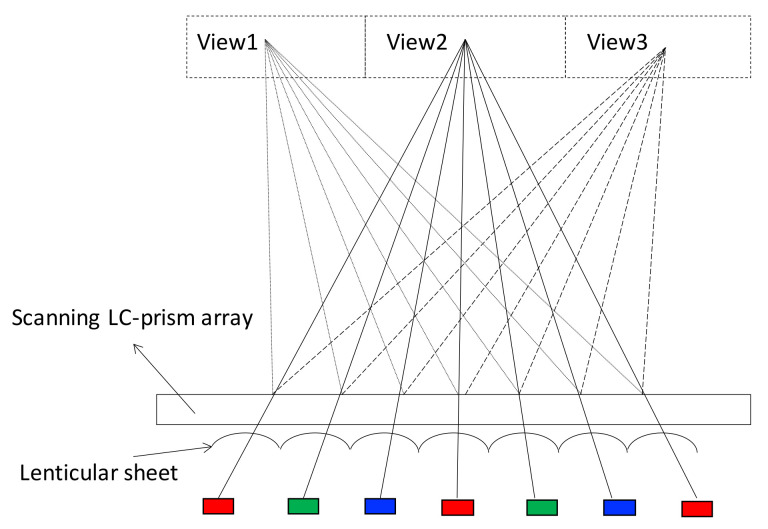
ITRI proposed a scanning LC-prism array to bend the light to different directions sequentially and realized a time-multiplex display [45].

Besides the above directional light guide, ITRI proposed a method with electro-controlled LC deflecting prisms to scan the projecting direction of light [45]. Figure 7 gives the configuration that the scanning LC-prism array is placed on the optical path to change the direction of light with time. In this case, a lenticular sheet is adopted to converge the rays from all the pixels to a single view, so each view is full resolution.

**Figure 8:**
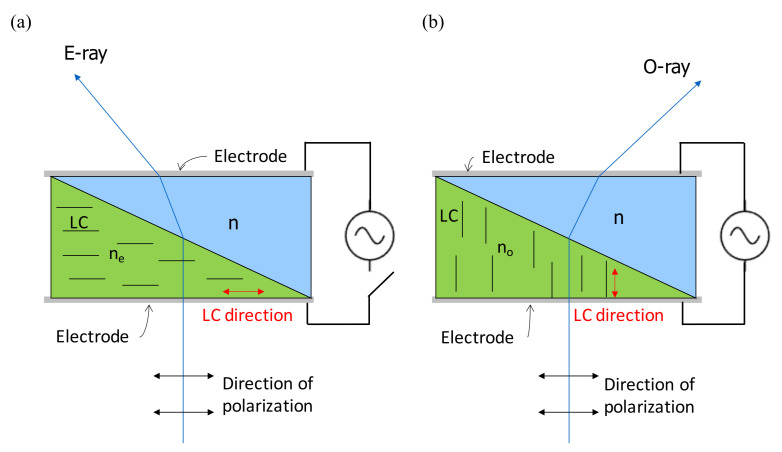
(**a**) Without applying an electric field, the LC lies down and behaves as $n_{e}$, and the ray is bent to left owing to $n_{e}>n$. (**b**) By applying an electric field, the LC stands up and behaves as $n_{o}$ that bends the ray to the right due to $n_{o}<n$ [45].

In Figure 8, the scanning prism is configured with an isotropic prism with refractive index n and a LC-filled prism whose refractive index is determined by the orientation of LC. Without applying an electric field, the LC direction is parallel to the polarization of light, and it behaves as refractive index of $n_{e}$; by applying an electric field, the LC direction is perpendicular to the polarization of light, and it behaves as a refractive index of $n_{o}$. If $n_{e}>n>n_{o}$, then the light is bent to left without applying an electric field and bent to right with applying an electric field. Thus, the direction of light is controlled by the applying electric field. By scanning the light direction, time-multiplex multiview display was realized.

## 5. Integral Imaging

**Figure 9:**
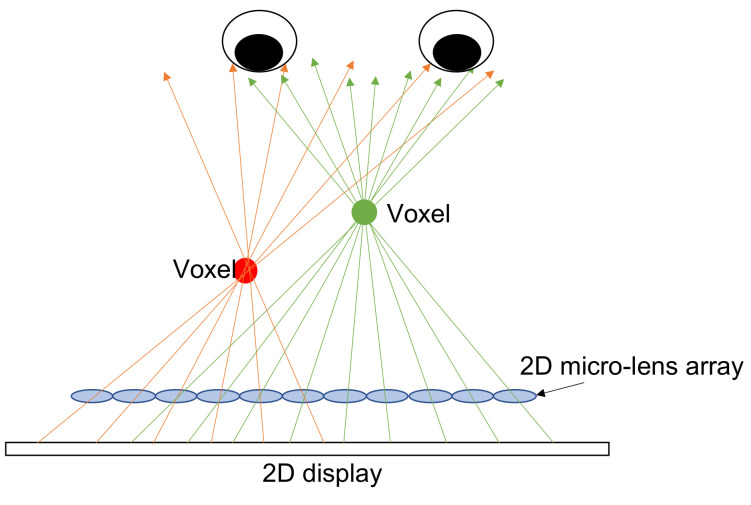
The principle behind integral imaging: The light field is reconstructed by the rays from the 2D display, which are directed to the voxel’s position by the micro-lens 2D array and intersect with a voxel.

Integral imaging (InIm) was invented by the Nobel laureate in physics, Gabriel Lippmann in 1908, and he coined this technique as “integral photography” [46]. Figure 9 shows the idea behind InIm: A voxel [2] is formed by intersecting rays being emitted from the 2D display and being directed to the position of voxel by the 2D micro-lens array. In other words, the multiple perspectives of a voxel are recorded by the 2D micro-lens array and reconstructed by the reversed way, and it presents a full parallax. If the ray density is high enough to allow at least two rays into a single eye, then it shows the accommodation effect [47] that resolves the accommodation-convergence conflict [6]. Hence, it mitigates the fatigue problem. The range of pupil diameter is 4–6 mm [48], indicating the ray separation is 2–3 mm when arriving at the eyes, so hundreds of rays in horizontal and vertical directions are required to cover a head movement in hundreds of millimeters, and the overall ray number could be tens of thousands; thus, the native display resolution must be tens of thousands-fold the perceived resolution, so an extremely-high-resolution display is required.

A large-screen InIm of 87 inch and a 360 light-field display with a holographic functional film was demonstrated [49,50]. In addition, a floating 96 × 96 view-point system was demonstrated at a 45 viewing angle [51]. Another interesting application of InIm is tabletop displays. A 360 interactive tabletop display comprised of an 8K LCD, lens array, optical diffuser film, and a hand-tracking device has been demonstrated [52].

## 6. Electronic Holography

**Figure 10:**
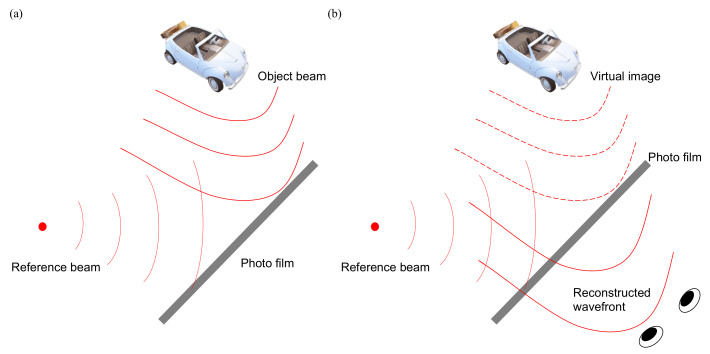
(**a**) The wavefront of the object beam interferes with the reference beam, and the fringe is recorded on a photo film to form a hologram. (**b**) Illuminating a hologram with the original reference beam reconstructs the wavefront of the object beam, so it includes the phase and amplitude such that eyes sense the depth of the object.

Holography was invented by the Nobel laureate, Dennis Gabor [53] in 1947. It records all the information of the light field, consisting of its amplitude and phase. In a reversed manner, the light field is reconstructed as a 3D image. Since holography does not mimic 3D with 2D images, it builds the authentic 3D wavefront instead; hence, holography is regarded as the ultimate 3D display. The formation of holography, in Figure 10, is that the object beam interferes with the reference beam coherently and the interference fringe is recorded on a photo film. Since the fringe carries the phase and amplitude information of the object beam, we can reconstruct the wavefront of object beam in reversal by illuminating the hologram with the original reference beam, so eyes perceive not only the amplitude but also the phase that produces the sensation of depth.

Electronic holography is an ideal solution for autostereoscopic display; however, it faces some large challenges. First, an ultrahigh resolution spatial light modulator (SLM) is required. The fringe width could be one half the wavelength, so it can go down to 200 nm based on 400 nm blue light; then, a 127,000-ppi SLM with 200-nm pixel size is needed to display such a narrow fringe. So far, the commercial SLM can achieve only 7000 ppi [54], so it needs a huge jump in pixel density. Second, to compute, restore, and display such a high resolution image, a huge computing power, transfer rate, and memory are required [55].

The Media Lab at Massachusetts Institute of Technology (MIT) developed an electronic holographic display, Mark III [56]. It utilized a lithium niobate guided-wave acousto-optic device as an SLM that provides 200 MHz bandwidth to create the fringe pattern, which can display a 24 viewing angle, an 80 mm × 60 mm × 80 mm viewing volume with a 532 nm laser source. University of Arizona developed a updatable holographic display with a photorefractive polymer as an SLM, which exhibits a viewing angle of 45, 4 inches; the downside is that it takes two seconds to refresh a frame [57,58]. ITRI proposed another configuration of a head-mounted holographic display that reduces the required pixel density to the level of presentation of the SLM [59,60]. For this head-mounted configuration, the holography display only needs to generate a small viewing angle around 4 to cover a single eye; thus, the fringe width is around a few microns, which can be displayed in the present SLM.

## 7. Comprehensive Analysis of 3D Display Based on LCD, OLED, and Micro-LED

Nowadays, LCD and OLED dominate the display market, and the micro-light emitting diode (Micro-LED) is the next emerging display technology. All of them can be used to implement 3D displays based on different scenarios. LCD requires a backlight to light the display on, so it has an extra flexibility to implement the light-directional-control elements within the backlight, such as the parallax barrier in Figure 1b, directional backlight [61,62], or a light-bar method introduced in Section 8. However, LCD has a slow response time around few ms [63,64], so it is hard to achieve a high refresh rate. On the contrary, OLED and Micro-LED both achieve fast response times of the order of µs and ns, respectively [65]. Hence, they are beneficial to time-multiplex. Another drawback of LCD is that it is less power efficient than OLED and Micro-LED; using a parallax-barrier setup would further deteriorate the power efficiency.

**Figure 11:**
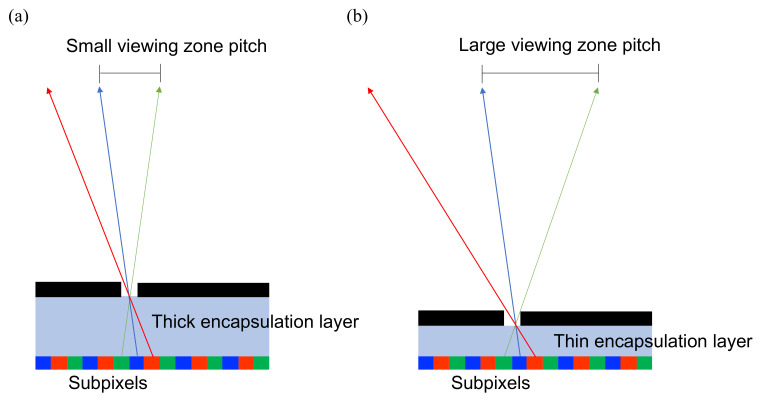
(**a**) The viewing zone pitch is small in a thick-encapsulation-layer system, such as LCD, that has a thick front glass around 0.5 mm. (**b**) The thin-encapsulation-layer system, e.g., OLED/Micro-LED has an ultra-thin film less than 1 µm, which shows a large viewing zone pitch.

As shown in Figure 11, LCD usually has an encapsulation glass around 0.5 mm thick, which leads to small viewing zone pitch comparing to the larger viewing zone pitch of OLED/Micro-LED whose encapsulation layer is less than 1 µm [66]. Under the same number of views, small viewing zone pitch shrinks the viewing angle, which is harmful to auditing experience. Hence, OLED/Micro-LED have the advantage of enlarging the viewing angle than LCD with the same number of views.

**Figure 12:**
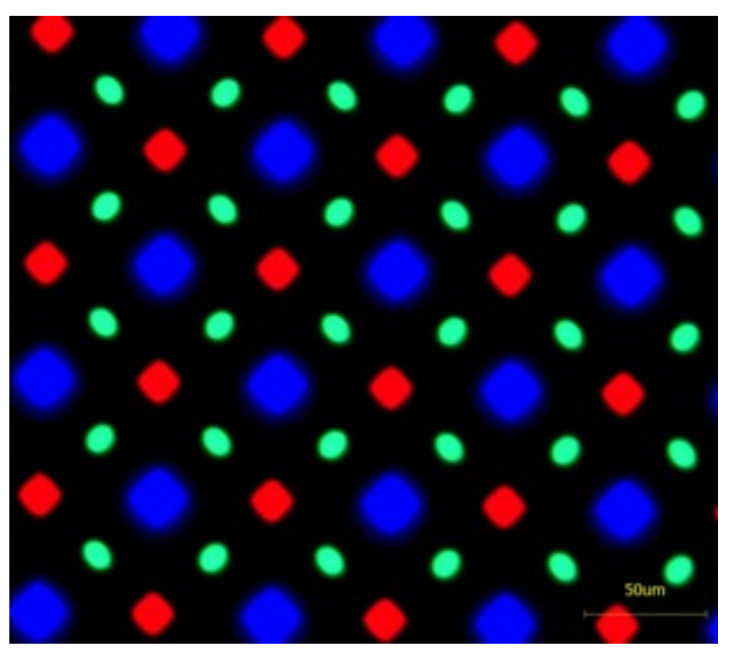
The pentile-pixel-arrangement image captured from an OLED display, showing a more sophisticated pattern than the RGB pattern of LCD, such that it is trickier to design the parallax barrier or lenticular pattern.

**Figure 13:**
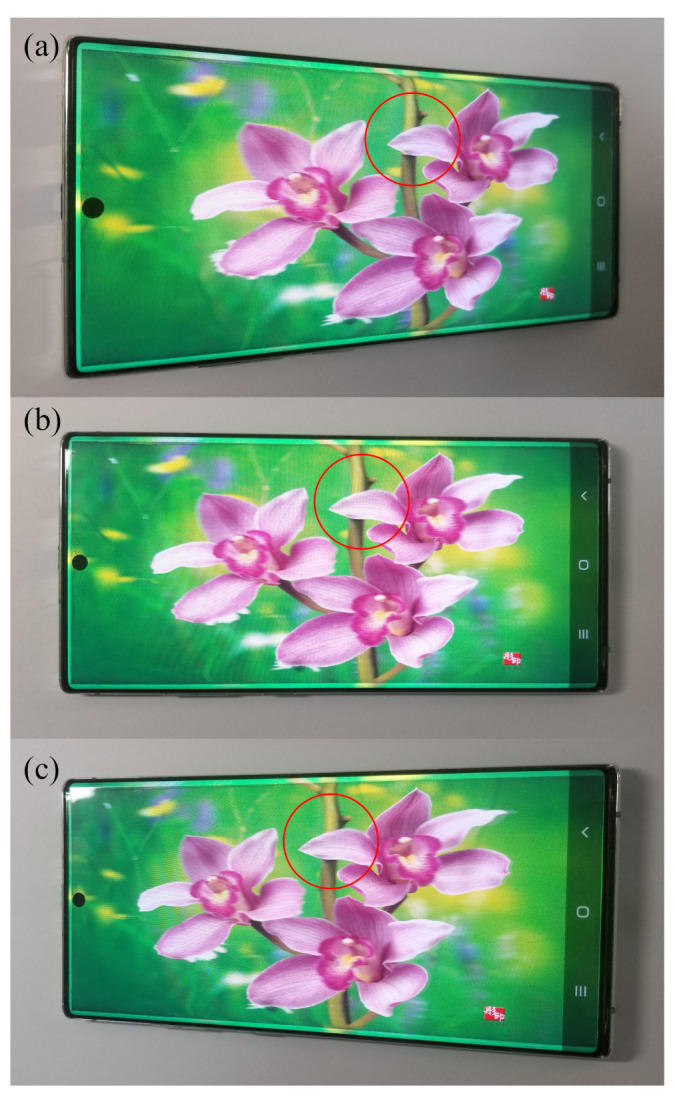
We have implemented an autostereoscopic display with an OLED smartphone. Comparing the (**a**) left, (**b**) middle, and (**c**) right views, the relative positions of petals and trunk (in the red circle) are different, and it shows a horizontal parallax.

On the other hand, OLED usually has a pentile-pixel arrangement (Figure 12), which is more sophisticated than LCD’s RGB arrangement, and it requires a special pattern design for the parallax barrier. Lee and Kim have shown how to design the parallax barriers in their work [67,68]. In our group, we also developed a slanted-barrier autostereoscopic display with OLED, whose slanted angle is arctan(1/4), owning a 12-view, 166-ppi, viewing angle up to 50, because we removed the cover glass and took the advantage of the thin encapsulation of OLED. The left, middle, and right perspectives are shown in Figure 13, and it shows the horizontal parallax that the relative positions of petals and trunk change over views.

Micro-LED is further superior to LCD/OLED with even lower power consumption and higher brightness [65]. The most impressive superiority is the ultra-high resolution as 8500-ppi [69] that can even support a super-multiview or InIm setup. OLED has achieved 10,000-ppi [70] as well, but its brightness is far lower than Micro-LED by at least one order. The overall comparison is summarized in Table 1.

## 8. The Future of 3D Display: Micro-LED Plays the Key Role

Micro-LED is an emerging technology that potentially drives the realization of 3D displays. Micro-LED provides the pixel size down to few microns, equivalent to tens of thousands of ppi [71]. Micro-LED satisfies the ultra-high-resolution requirement for InIm, and it is able to push the development of InIm greatly forward. Second, Micro-LED provides response time of nanoseconds such that it can be used as a time-multiplex display, so every time-multiplex method in Section 4 can be applied with Micro-LED. Third, Micro-LED offers a partial-coherent light source because of its small-area luminance, and it is capable of replacing the laser as the point light source to reduce the speckle effect for the electronic holographic display [72].

**Figure 14:**
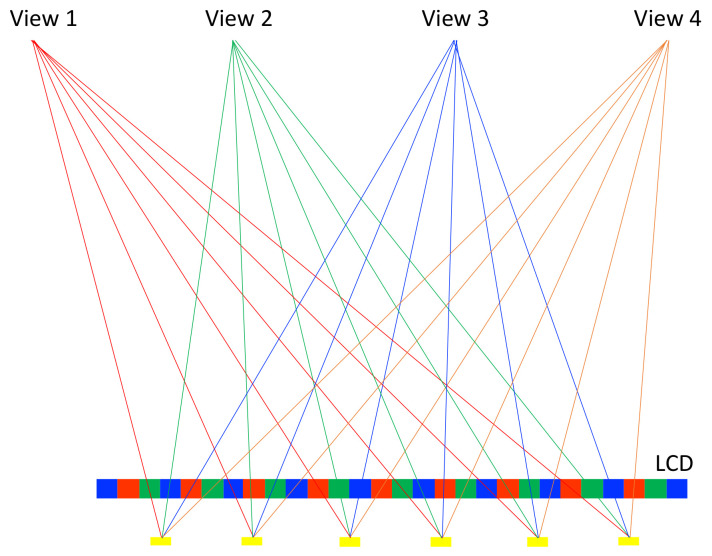
The Micro-LEDs form a light-stripe 1D array with a well-designed pitch, such that the partial pixels are projected to the corresponding views.

On the other hand, considering Micro-LED as a function of the backlight, the configuration in Figure 14 illustrates how the rays propagate through each pixel and converge to the viewing zone; here is an example for a four-view display, and the pitch of line stripes is slightly larger than four times the pixels. In this manner, the narrower the light stripe is, the less crosstalk is induced. Taking the advantage of small dimension of Micro-LED, the crosstalk can be effectively suppressed. ITRI [73,74] has developed a light-stripe backlight with inverted trapezoids, but its light efficiency is moderate. Now, it is possible to assemble Micro-LEDs as the light-stripe backlight and promote their light efficiency significantly.

**Figure 15:**
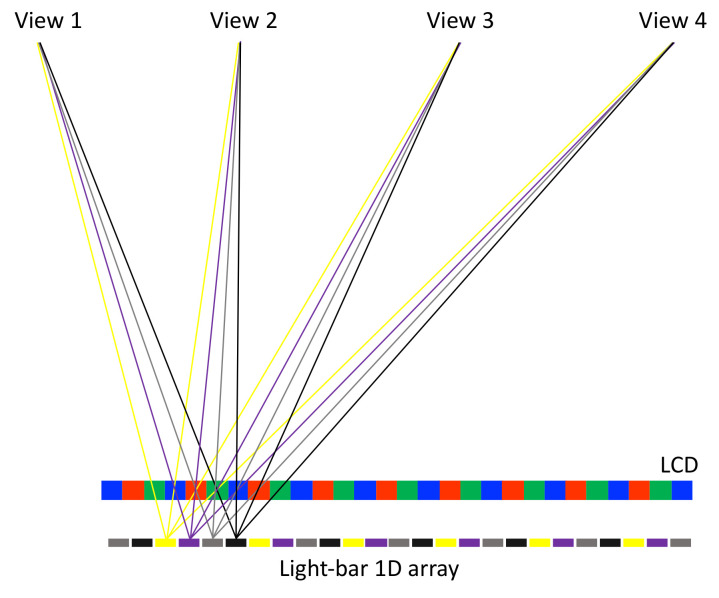
Introducing multiple sets of light-stripe arrays (grouped by colors) and switching each set of light stripes on and off sequentially allows every pixel to be perceived at each view. It is a time-multiplex method to achieve full resolution. Here, only the rays emitted from a single light-stripe set are plotted, and each view receives four pixels rather than a single pixel to demonstrate that a full-resolution image is perceived.

This light-stripe method can be generalized to time-multiplex display with multiple sets of light-stripe arrays. In Figure 15, four light-stripe sets are introduced, and they are manipulated with the lighting on and off in the order of yellow, purple, grey, and black cyclically. Each light-stripe set projects a quarter of the pixels to a single eye, and four sets of light stripes allow all the pixels to be perceived by a single eye. Hence, it achieves a full resolution with time-multiplex lighting on-and-off. For the four-view example, it turns 240 Hz lighting on and off. Based on the fast response of Micro-LED, it is easy to turn high-frequency lighting on and off. ITRI [45,61] has demonstrated another variant with light stripes and a lenticular sheet to collimate the backlight into a single direction; turning on different light-stripe sets directs the collimated backlight into different directions, and full-resolution images are delivered into various directions.

In addition, Micro-LED can be transferred onto a transparent glass substrate, and it becomes a transparent display that owns a floating 3D effect [75]. Japan virtual idol Hatsune Miku already demonstrated a projection image onto a transparent curved screen to mimic a holographic image [76]. Furthermore, Micro-LED has much higher luminance, up to tens of thousands of nits, than LCD, OLED, so it can be used outdoors where the ambient light is high.

Lastly, Micro-LED has a fast response time down to nano-seconends [65], it is especially suitable for time-multiplex 3D displays. Speeding up the frame rate up to 960 Hz and with the same-speed scanning barrier or a deflector such as the LC deflector shown in Figure 8, it generates a sixteen-fold multiview without any loss of resolution. Moreover, Micro-LED is more power-saving such that it prolongs the battery time for mobile devices, such as smart phones and tablets.

## 9. Conclusions

We comprehensively reviewed the understanding of how human eyes physiologically perceive three-dimensional objects, and the optical principles behind autostereoscopic displays, including the concept of the light field, parallax barrier, lenticular lens, integral imaging, and electronic holography. On top of that, based on LCD, OLED, and Micro-LED, we investigated their pros and cons for the implementation of autostereoscopic displays. Among these technologies, Micro-LED has the advantages of high resolution, fast response time, ultra-thin encapsulation layer, and high brightness, and they are beneficial to improving the performance of autosteroscopic displays. Based on these features, we proposed several implementations of autostereoscpic displays with Micro-LED: a Micro-LED light-stripe backlight with a LCD, a high-resolution Micro-LED display with a micro-lens array or a high-speed scanning barrier/deflector, and a transparent floating display.

## Figures and Tables

**Table 1:** The pros-and-cons comparison of LCD, OLED, and Micro-LED for 3D-display applications.

Type	Pros for 3D Display	Cons for 3D Display
LCD	1. LCD is more flexible regarding installation of the light-directional-control element cooperating with the backlight or in front of the color filter.	1. Slow response time (∼ms), unsuitable for time multiplex. 2. Cover glass is ∼0.5 mm thick, leading to a small viewing zone pitch. 3. Moderate brightness (∼1000 nits), unfavorable for the parallax-barrier setup.
OLED	1. High resolution ∼10,000-ppi compensates for the resolution loss of multiview. 2. Fast response time (∼μs), suitable for time-multiplex. 3. Ultra-thin encapsulation layer, leading to a larger viewing zone pitch.	1. No backlight, the light-directional-control element must be mounted in front of the panel, less flexible setup. 2. Moderate brightness (∼1000 nits), unfavorable for parallax barrier.
Micro-LED	1. High resolution of ∼8500-ppi compensates for the resolution loss of multiview. 2. Fast response time (∼ns), suitable for time multiplex. 3. Ultra-thin encapsulation layer <1 μm, leading to a larger viewing zone pitch. 4. High brightness (∼10,000 nits) and low power consumption (6∼7 cd/W) compensates for light loss of the parallax barrier.	1. No backlight, the light-directional-control element must be mounted in front of the panel, less flexible setup.

## Data Availability

Not applicable.
